# Susceptibility-Weighted Imaging (SWI): Technical Aspects and Applications in Brain MRI for Neurodegenerative Disorders

**DOI:** 10.3390/bioengineering12050473

**Published:** 2025-04-29

**Authors:** Federica Vaccarino, Carlo Cosimo Quattrocchi, Marco Parillo

**Affiliations:** 1 Radiology, Multizonal Unit of Rovereto and Arco, APSS Provincia Autonoma Di Trento, 38123 Trento, Italy; carlo.quattrocchi@unitn.it (C.C.Q.); marco.parillo@apss.tn.it (M.P.); 2 Research Unit of Diagnostic Imaging and Interventional Radiology, Department of Medicine and Surgery, Università Campus Bio-Medico di Roma, 00128 Rome, Italy; 3 Centre for Medical Sciences-CISMed, University of Trento, 38122 Trento, Italy

**Keywords:** radiology, neurology, susceptibility-weighted imaging, quantitative susceptibility mapping, magnetic resonance imaging, brain diseases, multiple sclerosis, Lewy body dementia, Parkinson disease, cerebral amyloid angiopathy

## Abstract

Susceptibility-weighted imaging (SWI) is a magnetic resonance imaging (MRI) sequence sensitive to substances that alter the local magnetic field, such as calcium and iron, allowing phase information to distinguish between them. SWI is a 3D gradient–echo sequence with high spatial resolution that leverages both phase and magnitude effects. The interaction of paramagnetic (such as hemosiderin and deoxyhemoglobin), diamagnetic (including calcifications and minerals), and ferromagnetic substances with the local magnetic field distorts it, leading to signal changes. Neurodegenerative diseases are typically characterized by the progressive loss of neurons and their supporting cells within the neurovascular unit. This cellular decline is associated with a corresponding deterioration of both cognitive and motor abilities. Many neurodegenerative disorders are associated with increased iron accumulation or microhemorrhages in various brain regions, making SWI a valuable diagnostic tool in clinical practice. Suggestive SWI findings are known in Parkinson’s disease, Lewy body dementia, atypical parkinsonian syndromes, multiple sclerosis, cerebral amyloid angiopathy, amyotrophic lateral sclerosis, hereditary ataxias, Huntington’s disease, neurodegeneration with brain iron accumulation, and chronic traumatic encephalopathy. This review will assist radiologists in understanding the technical framework of SWI sequences for a correct interpretation of currently established MRI findings and for its potential future clinical applications.

## 1. Introduction

Magnetic resonance imaging (MRI) has transformed medical diagnostics by enabling the detailed exploration of tissue properties through a range of contrast mechanisms that offer unique insights into tissue structure and pathology. Advanced techniques, such as susceptibility-weighted imaging (SWI) and quantitative susceptibility mapping (QSM), have further expanded the capabilities of MRI. SWI, by integrating high spatial resolution with a 3D gradient recalled echo (GRE) sequence and applying a phase mask, enhances the visualization of paramagnetic and diamagnetic substances and excels at detecting microstructural abnormalities like iron deposition, microbleeds, and venous anatomy [1]. On the other hand, QSM holds promise for quantitative assessments of magnetic susceptibility, providing an image that helps distinguish hemorrhage from calcification and mitigates the negative effects seen in SWI during the reconstruction process [2].

Despite their potential, these techniques generate complex contrasts that are often difficult to interpret. A thorough understanding of the underlying physics and the subtle tissue variations they reveal is crucial for accurate analysis. The concept of SWI originated from the observation that bulk magnetic susceptibilities influence local frequency shifts, analogous to chemical shifts in spectroscopy, but here the effect depends on the geometry of the object [3].

Brain MRI is the primary non-invasive imaging technique currently used in clinical practice for the study of the central nervous system [4,5,6,7], where SWI has established itself as a widely utilized clinical tool for imaging deoxyhemoglobin in veins, iron deposition, hemorrhages, calcifications, and microbleeds. Among the various pathologies of the central nervous system, in recent years there has been an increase in neurodegenerative diseases due to the aging of the population [8]. Neurodegenerative diseases encompass a spectrum of disorders characterized by the progressive degeneration and dysfunction of the nervous system, particularly affecting neurons, and often leading to cognitive, motor, and functional impairments, with symptoms varying across the range of severity and progression [9]. Given the difficulty in interpreting brain MRI in cases of suspected neurodegenerative disease with unclear clinical presentations, innovative imaging techniques such as SWI are being explored for their potential to improve early detection and ongoing patient care.

This narrative review explores the technical underpinnings of SWI and examines its less conventional uses in the context of neurodegenerative diseases, where it often remains underutilized in clinical protocols and its full potential is not yet fully integrated into diagnostic workflows. By synthesizing research up to the latest findings, we aim to illuminate the wider potential of this MRI sequence in clinical practice, providing up-to-date data to address everyday diagnostic challenges.

## 2. Materials and Methods

This review was prepared through a comprehensive literature search using established academic databases such as PubMed, Scopus, and Web of Science to identify relevant studies. The literature search, which included articles published up to December 2024, resulted in the selection of 75 articles, including review articles, original research papers, and case reports, published between 1992 and 2024, with the exception of one article published in 1968. Keywords used in the search included combinations of “SWI”, “susceptibility-weighted imaging”, “neurodegenerative diseases”, “technical aspects”, “magnetic resonance imaging (MRI)”, “iron deposition”, and “brain imaging”. Boolean operators such as “AND”, “OR”, and “NOT” were applied to refine the search results. We included papers that discuss technical aspects of SWI and its applications in brain MRI for neurodegenerative disorders. Studies published in languages other than English were excluded.

The nomenclature of sequences designed to detect changes in magnetic susceptibility varies depending on the MRI manufacturer. For instance, Siemens refers to this sequence as SWI, while GE names it susceptibility-weighted angiography (SWAN). Philips uses the term SWI-phase (SWIp), Hitachi designates it as blood-sensitive imaging (BSI), and Canon refers to it as flow-sensitive black blood (FSBB). To maintain clarity, in this review we will use the term “SWI” when referring to these sequences in general, before delving into the specific technical differences among them [10]. The paper is organized into two primary sections: the first provides a synthesis of the key technical aspects of the methods employed, while the second focuses on their application in major neurodegenerative disorders, specifically addressing relevant imaging signs and findings.

## 3. Technical Fundamentals of SWI

Magnetic susceptibility (χ) is a physical characteristic that defines how a substance responds magnetically when exposed to an external magnetic field, such as the field strength (B0) of an MRI scanner. Variations in tissue magnetic susceptibility (Δχ) lead to local magnetic field differences (ΔB), affecting the MRI signal and creating image contrast [11]:

ΔB = Δχ ⋅ B0

In biological systems, substances are generally categorized as paramagnetic or diamagnetic, with the latter exhibiting a negative χ that slightly reduces the overall magnetic field strength; most biological tissues, including lipids, water, calcium, myelin, and various proteins, are predominantly diamagnetic [12,13,14]. Paramagnetic substances, instead, which possess a positive χ, influence the MRI signal’s precession frequency in a manner opposite to diamagnetic materials and are typically associated with unpaired electrons in elements like copper and iron [14,15]. Variations in χ within tissue affect the MRI signal in two key ways: (1) reducing signal magnitude by increasing the transverse relaxation rate, observable as hypointense signal on T2 and T2*-weighted images or hyperintense signal on R2 and R2* maps; and (2) altering signal phase, which can be positive or negative depending on the voxel’s bulk χ [15,16]. The MRI signal of a voxel reflects the transverse magnetization, representing the combined magnetic moments of hydrogen protons within the voxel, with signal intensity determined by the magnetization vector’s magnitude and its phase defined by the accumulated angle relative to a reference axis. In a GRE sequence, the MRI signal’s magnitude and phase are influenced by χ, with magnitude decay over time caused by T2* effects, which result from spin mobility and χ-induced distortions [17]. Consequently, the hypointense signals on T2*-weighted images suggest the presence of substances with χ distinct from that of water. For quantitative assessment, most scanners generate R2* maps (where R2* = 1/T2*) using multiecho gradient echo sequences and applying a monoexponential model to the signal decay as a function of echo time (TE). For practical reasons, R2* mapping is often chosen over T2* mapping. This is because R2* maps provide a more straightforward visualization of χ-induced signal changes, with the foci of interest appearing as areas of increased signal intensity [1,18]. However, while magnitude data cannot differentiate between diamagnetic and paramagnetic effects, phase evolution over time can, with the appearance of diamagnetic substances on phase images—bright or dark—depending on the scanner’s magnetic field direction (right- or left-handed systems), where left-handed systems show paramagnetic substances as bright and strongly diamagnetic ones as dark.

SWI is derived from GRE pulse sequences, leveraging their inherent sensitivity to variations in tissue χ. This sensitivity arises from the fact that GRE sequences do not refocus spins dephased by magnetic field inhomogeneities. The core mechanism driving SWI lies in the bulk χ of the tissue, enabling the technique to detect even subtle variations in the tissue’s magnetic composition with exceptional precision [19]. Traditional T2*-weighted GRE sequences have been widely used to identify iron deposits, blood products, and calcifications. However, modern SWI sequences incorporate advanced techniques and optimizations that offer greater sensitivity and improved detection capabilities [20]. SWI sequences are no longer acquired in 2D mode, but are typically obtained in 3D mode, enabling the use of smaller voxel sizes and thinner slices for enhanced spatial resolution. Flow compensation is applied in all three directions to minimize artifacts, and parallel imaging techniques are employed to shorten acquisition times [21]. Within a single repetition time (TR) interval, either single or multiple echoes can be collected. A defining characteristic of SWI is the independent processing and display of magnitude and phase data, which can also be combined for improved diagnostic accuracy [22]. The recommended parameters are typically TR of 25–50 ms, TE of 20–40 ms, and flip angles ranging from 15° to 20°. As field strength increases, shorter TR/TE values and smaller flip angles are generally used to optimize image quality.

**Figure 1:**
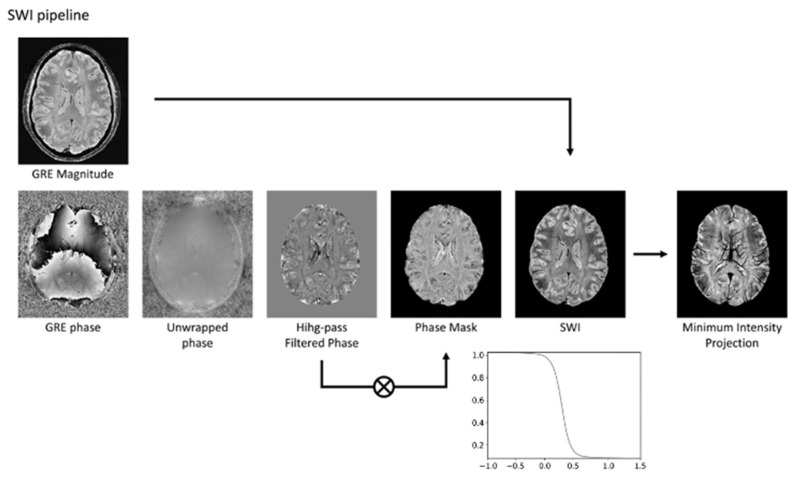
The SWI pipeline involves several steps. Initially, the phase image undergoes unwrapping to correct for phase discontinuities. Subsequently, a high-pass filter is applied to the unwrapped phase to eliminate phase variations not associated with tissue properties. The filtered phase data is then transformed into a phase mask, typically using a sigmoid function. Finally, the magnitude image is multiplied by this phase mask to generate the SWI. To enhance vein visualization, a minimum intensity projection technique can be employed. Modified from Rimkus et al. [18] under the terms and conditions of the Creative Commons Attribution (CC BY) 4.0 license (https://creativecommons.org/licenses/by/4.0/ (accessed on 1 March 2025)).

As shown in Figure 1, SWI-phase images undergo high-pass filtering to eliminate phase contributions unrelated to tissue, such as background fields and field inhomogeneities. These filtered phase images are then scaled to a range of 0 to 1 using a defined scaling function, where a scaled value of 1 conventionally represents a negative phase variation. The final SWI image is created by voxel-wise multiplication of the phase mask with the magnitude image [1]. This processing technique optimizes susceptibility contrast within SWI images, rendering even minor vessels as regions of pronounced signal void. This enables the acquisition of MRI venography by applying a minimum intensity projection (MinIP) reconstruction to thicker image slices (Figure 1).

**Figure 2:**
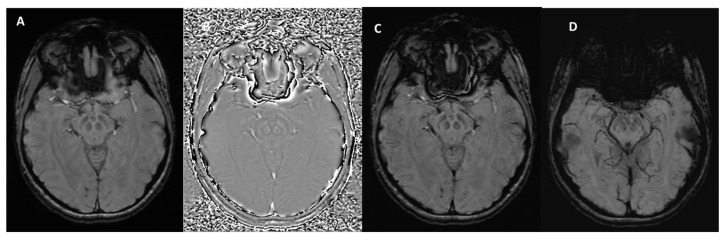
SWI outputs from the Siemens MAGNETOM Aera (Erlangen, Germany) 1.5T MRI scanner at the level of the midbrain. (**A**) Magnitude Image: displays the intensity of the signal, showing general tissue contrast. (**B**) Phase Image: highlights variations in magnetic susceptibility, useful for detecting iron deposition and microhemorrhages. (**C**) SWI-Processed Image: combines magnitude and phase data to enhance the visualization of microvascular structures and pathological features. (**D**) Minimum Intensity Projection: a multislice projection image that accentuates low-signal structures such as veins and calcifications for improved spatial visualization.

In summary, magnitude and phase data are reconstructed separately. The magnitude image, which highlights background tissue with a spin-density-like contrast, is preserved for diagnostic evaluation. In contrast, raw phase data require further processing to be clinically useful, typically involving the removal of low-frequency gradients and artifacts through high-pass filtering and local corrections. The result is a filtered phase image, ready for diagnostic use (Figure 2) [22].

### How Can Calcifications and Blood Be Differentiated on an SWI Phase Map?

Contemporary SWI sequences generate filtered phase images in which calcifications (diamagnetic) exhibit signal intensities that are the inverse of those associated with blood products (paramagnetic).

**Figure 3:**
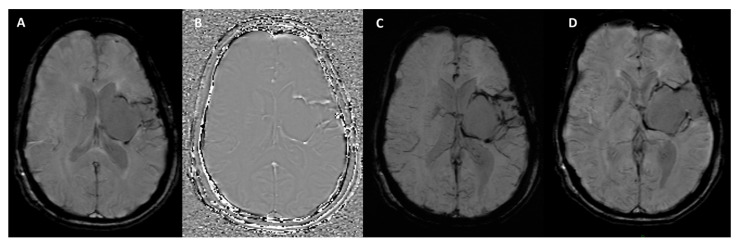
SWI sequence outputs from the Siemens MAGNETOM Aera (Erlangen, Germany) 1.5T MRI scanner in a patient with hemosiderin deposits surrounding a surgical cavity in the left cerebral hemisphere. (**A**) Magnitude Image: displays areas of hypointense signal indicative of hemosiderin deposits. (**B**) Phase Image: shows the hemosiderin deposits as hyperintense structures, highlighting their magnetic susceptibility effects. (**C**) SWI-Processed Image: combines magnitude and phase data to enhance visualization of susceptibility artifacts, such as the hemosiderin rim around the surgical cavity. (**D**) Minimum Intensity Projection: highlights the distribution of low-signal susceptibility-related structures, improving the visualization of hemosiderin deposits and venous anatomy.

**Figure 4:**
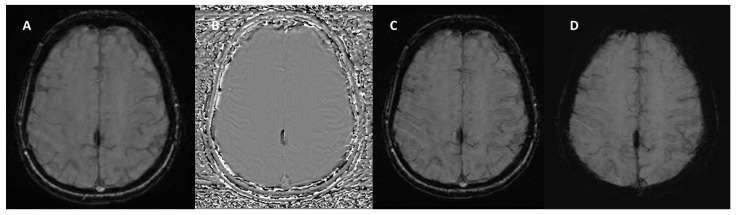
SWI sequence from the Siemens MAGNETOM Aera (Erlangen, Germany) 1.5T MRI scanner in a patient with calcification of the falx cerebri. (**A**) Magnitude Image: displays the calcification as a hypointense structure. (**B**) Phase Image: the calcification appears hypointense, consistent with its diamagnetic properties. (**C**) SWI-Processed Image and (**D**) Minimum Intensity Projection enhance the contrast of calcified structures and highlight low-signal calcification.

**Figure 5:**
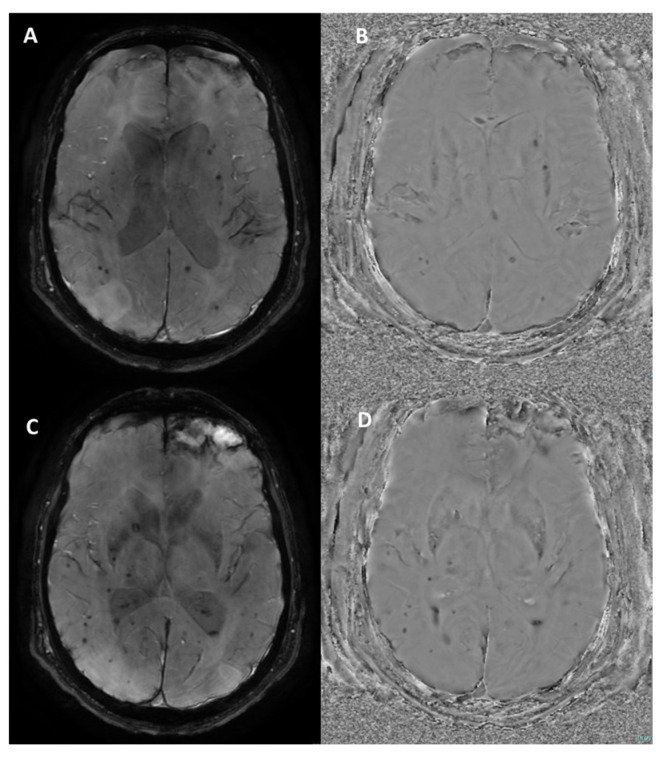
Susceptibility-weighted angiography (SWAN) sequence outputs from the GE Optima MR450w (Chicago, Illinois) 1.5T MRI scanner in a patient with diffuse axonal injury and a left frontal parenchymal contusion. (**A**,**C**) Magnitude Images: show multiple hypointense foci scattered throughout the brain parenchyma, consistent with microbleeds due to diffuse axonal injury, along with a larger hypointense area in the left frontal region indicative of a post-traumatic contusion. (**B**,**D**) Phase Images: the microbleeds and contusion appear hypointense, as is typical on GE systems. These findings are characteristic of traumatic brain injury, highlighting both diffuse and focal patterns of injury.

It is important to note that the appearance of blood and calcium on SWI phase images varies depending on the scanner manufacturer. Siemens and Canon employ “left-handed” reference schemes, causing blood products to appear bright (Figure 3 and Figure 4), while GE and Philips use “right-handed” references, where blood products appear dark (Figure 5).

## 4. SWI Applications in Neurodegenerative Disorders

The dynamics of iron deposition in the brain are characterized by both age-related increases and regional variations. A rapid rise in iron occurs in the lentiform nuclei, red nuclei, and substantia nigra during the first two decades. Subsequently, iron accumulation plateaus in the globus pallidus and putamen by the fifth decade, while the red nucleus and substantia nigra continue to accumulate iron. The cortex maintains low iron concentration in normal conditions [23]. In the context of neurodegenerative diseases, increased iron deposition and/or microbleeds are frequently observed in different parts of the brain [24]. With the rising average age of the global population, the prevalence of neurodegenerative disorders has significantly increased [8]. This demographic shift underscores the growing importance of refining diagnostic approaches to meet the challenges of these conditions. Evaluating brain scans for potential neurodegenerative disorders can be difficult, particularly when clinical presentations are vague. In these cases, imaging results may also be unclear and difficult to interpret, and SWI offer promising potential to support clinicians in both early detection and ongoing management. In the following sections, we delve deeper into the major applications of SWI in prevalent neurodegenerative diseases, including Parkinson’s disease (PD), atypical parkinsonian syndromes and Lewy body dementia, multiple sclerosis (MS), cerebral amyloid angiopathy (CAA), amyotrophic lateral sclerosis, Huntington’s disease, cerebellar ataxias, and chronic traumatic encephalopathy [25,26].

Alzheimer’s disease (AD) is the most common neurodegenerative disorder and some studies have demonstrated that AD patients predominantly present a lobar distribution of cerebral microbleeds, potentially correlated with cerebrospinal fluid levels of amyloid-beta and phosphorylated tau 181 protein [27]. Nevertheless, SWI is primarily used in routine diagnostic workflow in differentiating AD from dementia with Lewy bodies and CAA, where more specific imaging features, as discussed below, may be detected [23]. Furthermore, although prion disease is part of the neurodegenerative disorders, since it does not present characteristic signs in SWI, it is not discussed further.

### 4.1. Parkinson’s Disease, Lewy Body Dementia, and Atypical Parkinsonian Syndromes

**Figure 6:**
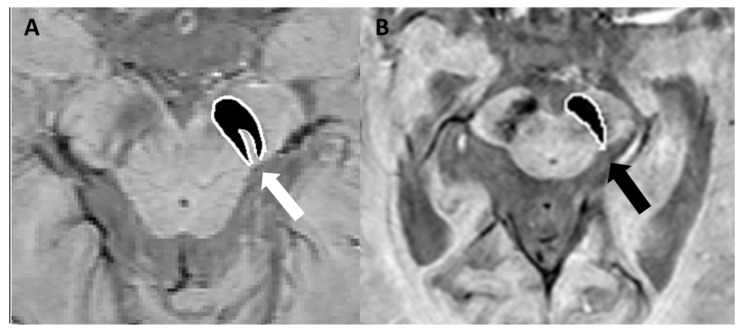
(**A**) SWI sequence acquired with a 3T scannerat the level of the mesencephalon, demonstrating the characteristic “swallow-tail sign” (white arrow). (**B**) Loss of the “swallow-tail sign” in a patient with Parkinson’s disease; the black arrow indicates the absence of the normal hyperintense signal in the substantia nigra. Modified from Lee et al. [37] under the terms and conditions of the Creative Commons Attribution (CC BY) 4.0 license (https://creativecommons.org/licenses/by/4.0/ (accessed on 1 March 2025). Image B has been magnified by 350% compared to the original. Accumulations of Lewy bodies are a pathological hallmark observed in both dementia with Lewy bodies and PD. The distinction between PD and dementia with Lewy bodies remains a subject of debate, with some researchers proposing they represent a spectrum of the same disease process. Dementia with Lewy bodies is diagnosed when cognitive impairment is the primary symptom or appears within 12 months of motor symptom onset. Conversely, PD is the diagnosis when motor features are dominant, though symptoms overlap and evolution occur over time in both conditions [38]. In this scenario, SWI can be used to differentiate AD from dementia with Lewy bodies based on the presence or absence of the swallow-tail sign, typically preserved in AD but not in dementia with Lewy bodies [36]. The abnormal findings observed in nigrosome-1 imaging lack the specificity needed to differentiate PD from atypical parkinsonian syndromes, including multiple system atrophy (MSA, parkinsonian and cerebellar subtypes), progressive supranuclear palsy (PSP), and corticobasal degeneration [39] where other MRI abnormalities may help in the differential diagnosis [1]. Compared to PD and PSP, MSA-parkinsonian type is characterized by greater iron deposition and atrophy within the posterolateral putamen in SWI sequences [40]. The distribution of iron deposition in SWI differs between PSP and PD. In PSP, iron accumulation is most prominent within several deep brain nuclei, including the putamen, red nucleus, substantia nigra pars reticulata, and cerebellar dentate nucleus, making it a distinguishing feature among atypical parkinsonian syndromes [41]. Finally, nigrosome-1 imaging is normal in both essential tremor and drug-induced parkinsonism, thus highlighting the role of SWI in the differential diagnosis with PD [1].

Following AD, PD is the second most prevalent neurodegenerative disorder, and it is characterized by motor symptoms resulting from the loss of dopaminergic neurons, which is associated with the accumulation of α-synuclein (Lewy bodies) and iron overload in the substantia nigra of the midbrain [28]. Indeed nigrosomes, a cluster of dopaminergic neurons within the pars compacta of the substantia nigra, are characterized by high neuromelanin content and low iron levels. They are divided into five regions known as nigrosomes 1 to 5 [29]. Nigrosome-1, the largest, plays a key role in PD due to significant dopaminergic neuron loss. On T2*-weighted or SWI sequences, nigrosome-1 appears as a hyperintense region. Optimal visualization is typically achieved with scanners of at least 3 Tesla field strength [30]. In 95% of healthy individuals, in fact, normal nigrosome-1 within the dorsolateral substantia nigra exhibits a characteristic “swallow tail” appearance [31,32]. In the literature, PD has been associated with a reduction in T2* signal within nigrosome-1 on T2*-weighted images. This phenomenon may arise from a reduction in neuromelanin levels within dopaminergic neurons, an accumulation of free iron that induces local magnetic field inhomogeneities and signal attenuation, or the disruption of iron–melanin complexes [33,34]. The absence of the “swallow-tail sign” demonstrates a diagnostic accuracy greater than 90% for PD and dementia with Lewy bodies, according to published reports [30,35,36] (Figure 6).

QSM is capable of highlighting elevated iron concentrations with high contrast in the substantia nigra and red nucleus during the initial phases of PD. This suggests QSM’s potential as an early diagnostic marker for PD. Moreover, QSM can illustrate the increasing iron levels as the disease progresses, a change that correlates with the advancement of PD [2,42]. Furthermore, QSM offers a potential method for distinguishing between MSA-parkinsonian type and PSP. This is because MSA-parkinsonian type is characterized by significant iron accumulation in the posterolateral putamen, while in PSP, the globus pallidus exhibits more prominent iron deposits [43,44].

Recent advancements highlight how radiomics tools can enhance the detection and characterization of nigrosome-1 loss in PD, overcoming variability in traditional imaging. Radiomic features derived from QSM demonstrate high accuracy, sensitivity, and specificity in differentiating PD patients from healthy controls [35]. Additionally, a study applying radiomics to both T1-weighted imaging and SWI demonstrated that combining features from these modalities significantly improves differentiation between PD, MSA, and healthy controls. In particular, the integration of texture features from the substantia nigra and shape features from the globus pallidus achieved high diagnostic performance, supporting the clinical utility of radiomics in distinguishing parkinsonian syndromes using routine MRI sequences [45]. Another study based on radiomic features from SWI magnitude images not only achieved good diagnostic performance in distinguishing PD from controls, but also identified features moderately correlated with Hoehn–Yahr stage, suggesting potential for assessing disease severity [46].

### 4.2. Multiple Sclerosis

MS is a significant autoimmune disease of the central nervous system, characterized by a range of debilitating symptoms resulting from inflammation, demyelination, and neurodegeneration [47,48]. Currently, the diagnosis of MS is largely dependent on MRI, which effectively illustrates the disease’s dissemination across both space and time as outlined by the 2010 McDonald criteria [49,50,51]. However, since the 2010 McDonald criteria have imperfect sensitivity and specificity, identifying additional radiological signs can be valuable in supporting the differential diagnosis from MS mimics [52,53].

**Figure 7:**
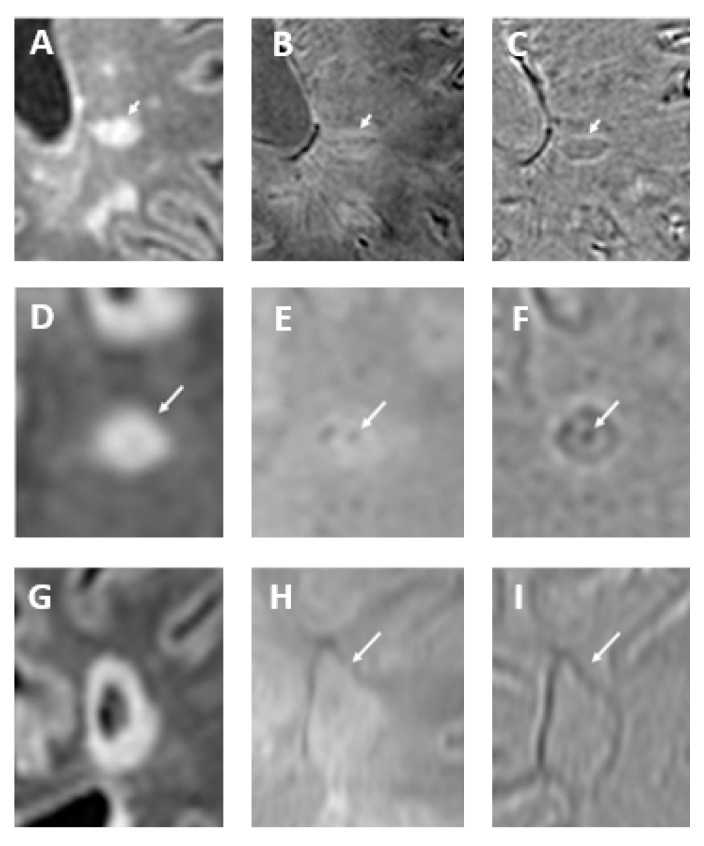
In oval-shaped lesions on fluid-attenuated inversion recovery (FLAIR) sequence (arrow in (**A**)), the central vein is visible as a hypointense line on SWI (arrow in (**B**)) and SWI-phase (arrow in (**C**)), creating a coffee-bean appearance. Conversely, in round-shaped lesions on FLAIR image (arrow in (**D**)), the central vein appears as a dark point on SWI (arrow in (**E**)) and frequently forms a target or doughnut shape on SWI-phase (arrow in (**F**)). Paramagnetic rim lesions are visible in both SWI and SWI-phase images. The FLAIR image (**G**) depicts a cavitated multiple sclerosis plaque, with a paramagnetic rim highlighted in both the SWI (arrow in (**H**)) and SWI-phase (arrow in (**I**)) images. Modified from Rimkus et al. [18] under the terms and conditions of the Creative Commons Attribution (CC BY) 4.0 license (https://creativecommons.org/licenses/by/4.0/ (accessed on 1 March 2025)).

Initially observed in high-field MRI studies [54], the “central vein sign” (CVS) is a radiological feature characterized by a detectable central vein within white matter lesions, exploiting the characteristic perivenular distribution of MS lesions and supports their venocentric origin [55,56]. It holds promise as an imaging biomarker for accurately differentiating MS from other white matter diseases of the central nervous system (e.g., gliosis, edema, and non-specific inflammation). A positive CVS is characterized by a thin, dark line or spot observed in at least two orthogonal planes, traversing the lesion centrally and equidistantly from its edges. To be eligible for evaluation, lesions must be distinct, non-overlapping, and measure at least 3–5 mm. One important factor to consider is that the shape of the lesion affects its appearance on CVS: oval lesions on fluid-attenuated inversion recovery (FLAIR) images typically exhibit a linear hypointense signal on SWI and SWI-phase, resembling a coffee bean; conversely, round lesions on FLAIR images usually demonstrate a punctate hypointense signal on SWI, often forming a target or doughnut shape on SWI-phase (Figure 7). To differentiate MS from other diseases using imaging, several threshold criteria have been proposed, including the “40% rule” (introduced by Evangelou et al. [56]), which distinguishes MS by identifying the burden of lesions with a central vein. To demonstrate the potential utility of this sign, a systematic review and meta-analysis published in 2019 identified an optimal threshold of 45% for the proportion of lesions exhibiting a central vein sign, showing a specificity of 99% and a sensitivity of 97% [57]. In addition to the CVS, emerging evidence links latent inflammation within chronic active MS lesions, characterized by the presence of iron-laden microglia visible as paramagnetic rim lesions on SWI, to both disease progression and neurodegeneration (Figure 7) [58]. Notably, this sign is visible in approximately half of patients diagnosed with either relapsing or progressive forms of MS [59,60]. Iron deposition in lesions changes over time. Initially, peripheral iron is observed in classic, reactive, slowly expanding chronic lesions. Progressive myelin and oligodendrocyte loss, spreading from the lesion’s edge to its center, results in nodular lesions with varying iron content in different compartments [61].

Recently, both the paramagnetic rim and the central vein sign have been incorporated into the 2024 update of the McDonald criteria [62].

QSM reveals high-contrast rims in chronic active MS lesions within the first 4 years, attributed to iron in activated microglia/macrophages. The later decrease in susceptibility makes serial QSM indicative of current inflammatory activity. Absence of increased susceptibility suggests acute or >5-year-old lesions, while its presence indicates an age of weeks/years, relevant for temporal dissemination [2].

### 4.3. Cerebral Amyloid Angiopathy

CAA is a vascular condition caused by the accumulation of amyloid-beta protein in the small- and medium-sized blood vessels of the brain’s cortex and surrounding leptomeninges, which can result in weakened blood vessels and fibrinoid necrosis. Beyond its association with hemorrhagic strokes, CAA is increasingly recognized for its connection to features typically considered hallmarks of neurodegeneration: brain atrophy and progressive cognitive decline. This association is observed even in brain regions unaffected by hematomas and in patients without new hemorrhagic strokes [25].

**Figure 8:**
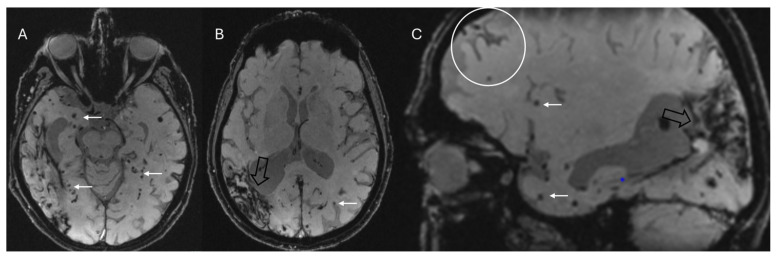
Multiple small lobar cortical and subcortical microbleeds (white arrows) on 3D-SWI acquired with a 1.5T scanner reconstructed both in axial (**A**,**B**) and sagittal (**C**) planes. Hemosiderin deposits from previous right parieto-temporal lobar hemorrhage (empty black arrows in (**B**,**C**)). Sagittal SWI reveals a coating along surface of the sulcus (cortical superficial siderosis, white circle in (**C**)). Note the absence of microbleeds in the basal ganglia in (**B**), which assists in the differential diagnosis with hypertensive microangiopathy. The combination of these findings allows for the diagnosis of probable cerebral amyloid angiopathy according to the Boston 2.0 criteria.

SWI, along with T2*-weighted sequences, is effective in detecting cerebral microbleeds, which are typically small (2–10 mm), multiple (≥2), and characterized by round or ovoid, homogeneously hypointense structures, primarily located in the frontal and parietal lobes (Figure 8) [63]. Multiple microbleeds at the gray–white matter junction (excluding the posterior fossa) strongly suggest the diagnosis of CAA, distinguishing it from hypertensive microhemorrhages, which typically occur in the basal ganglia, thalami, pons, and cerebellum [64]. Furthermore, the presence of convexity subarachnoid hemorrhage and cortical superficial siderosis on SWI can be a valuable clue in the diagnosis of CAA. Convexity subarachnoid hemorrhage involves bleeding limited to the cortical sulci of the brain’s convexity, while the ventricles, Sylvian fissures, and basal cisterns are generally spared [65]. Cortical superficial siderosis (Figure 8), a highly specific marker for CAA, is associated with an increased risk of future or recurrent intracranial hemorrhage and it appears as a hypointense, curvilinear rim that adheres to the cortex. Histological analysis typically reveals the presence of hemosiderin-laden macrophages. Although its exact origin remains under investigation, it is believed to be a sequel of a subarachnoid hemorrhage. Two distinct patterns are recognized: a focal form, involving fewer than four sulci, and a disseminated form, affecting four or more sulci [66]. A recent study comparing SWI and GRE-T2* in advanced CAA patients found that, while the overall detection rates for cortical superficial siderosis were similar (70.4% with SWI vs. 66.7% with GRE-T2*), SWI was significantly more sensitive in identifying the disseminated form—detected in 50% of cases compared to 37.04% with GRE-T2* (*p* = 0.008)—suggesting that SWI may provide additional advantages in assessing the extent and multifocality of cortical superficial siderosis [67].

According to the recent Boston 2.0 diagnostic criteria, CAA is probable in the presence of at least two strictly lobar hemorrhagic lesions (intracerebral hemorrhage, cerebral microbleeds, foci of cortical superficial siderosis, or convexity subarachnoid hemorrhage) on T2*-weighted or SWI sequences in the absence of any deep hemorrhagic lesions and other causes of hemorrhagic lesions [68].

### 4.4. Other Conditions

- *Amyotrophic lateral sclerosis* is a progressive neurodegenerative disease affecting upper and lower motor neuron function. While traditional MRI sequences struggle to reliably visualize axonal degeneration in the corticospinal tracts of amyotrophic lateral sclerosis patients, SWI has shown promise in revealing abnormalities in the motor cortex. Specifically, a low signal intensity region, termed the “motor band sign”, is observed more frequently in younger patients and may reflect iron deposition associated with upper motor neuron involvement [69]. The high contrast observed in the motor cortex via QSM may serve as a valuable and sensitive tool for both the diagnosis and prognosis of amyotrophic lateral sclerosis [2].
- *Hereditary ataxias* are characterized by the slow, progressive degeneration of the cerebellum and its pathways, resulting in motor incoordination and balance impairments. These impairments present as limb ataxia, gait and stance ataxia, dysarthria, and oculomotor signs. SWI imaging reveals atrophy of the cerebellar nuclei in spinocerebellar ataxia 6, Friedreich’s ataxia, and spinocerebellar ataxia 3 [70]. In patients with oculomotor apraxia, a key diagnostic indicator is the absence of the normal hypointensity in the dentate nucleus on 3T SWI and FLAIR scans, exhibiting both high sensitivity and specificity [71].
- *Huntington’s disease*, an autosomal dominant neurodegenerative disorder, involves the progressive loss of GABAergic neurons in the basal ganglia, notably the caudate and putamen (dorsal striatum). This neuronal loss leads to chorea, subcortical cognitive impairment, behavioral changes, and depression, typically beginning in midlife. Iron deposition within the basal ganglia (mainly globus pallidus) can sometimes manifest as decreased T2 signal and blooming on SWI [72].
- *Neurodegeneration with brain iron accumulation* refers to a diverse and progressive group of disorders characterized by excessive iron deposition in the brain, particularly within the basal ganglia, and the majority of these conditions have a genetic origin [73]. The “eye of the tiger” sign in the globus pallidus is the most recognized imaging feature, typically associated with pantothenate kinase-associated neurodegeneration (formerly known as Hallervorden–Spatz disease) [74]. However, it has been demonstrated that this sign is not pathognomonic, particularly in adult patients [75]. Caution is therefore needed to avoid misinterpreting this finding, as some authors have even reported that it may appear as a normal finding on 3T MRI scanners [76]. Furthermore, QSM shows promise in clearly visualizing age-atypical iron accumulation in the globus pallidus due to its high contrast capabilities [2,77].
- *Chronic traumatic encephalopathy* is characterized by perivascular accumulations of hyperphosphorylated tau in neurons and cellular processes, especially at the depths of the sulci. This neurodegenerative disease is associated with repeated head injuries, frequently encountered in contact sports [78]. Repetitive head impacts and traumatic brain injury can lead to microbleeds, which may have implications for the pathogenesis of chronic traumatic encephalopathy. Microhemorrhages associated with diffuse axonal injury frequently occur at the gray–white matter junction, in the corpus callosum, and within the brainstem. SWI can accurately identify these hemorrhagic foci, some of which may exhibit a linear configuration [79]. However, microbleeds have been observed infrequently in studies examining retired professional athletes from contact sports [80]. SWI has been shown to be more accurate and sensitive in detecting microbleeds associated with diffuse axonal injury compared to T2*-GRE sequences. Nevertheless, these findings have low specificity for chronic traumatic encephalopathy and may also be present in other traumatic brain injuries [79].

A summary of the main SWI findings and signs for each neurodegenerative condition can be found in Table 1.

## 5. Pitfalls and Limitations

**Figure 9:**
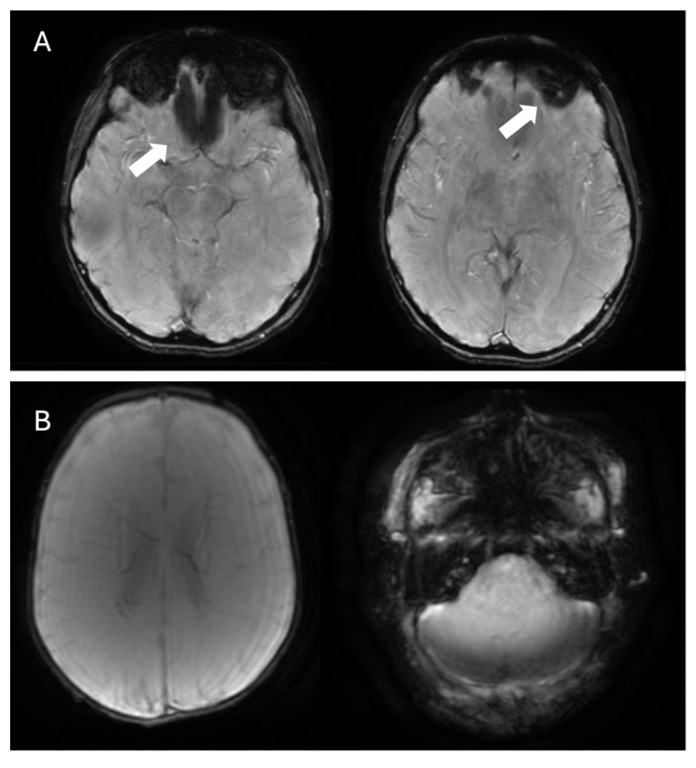
(**A**) Example of SWI acquisition showing artifacts affecting the region near the ethmoid air cells and the left orbit (white arrows). (**B**) Example of SWI acquisition with motion artifacts in a neonate.

Despite the numerous potential advantages of SWI, including its application in more unconventional settings, and considering its wide accessibility and integration into many routine MRI protocols, radiologists should remain mindful of its limitations. For example, while calcium deposits in the basal ganglia appear hypointense on SWI, their signal on phase images can be variable or even inverted, due to the presence of other elements such as iron. This overlap makes it difficult to clearly differentiate calcifications from hemorrhages. Additionally, SWI can be prone to artifacts, particularly at air–tissue interfaces, such as those near the paranasal sinuses or temporal bones, where magnetic susceptibility differences are more pronounced (Figure 9A). These artifacts can obscure adjacent anatomical structures or mimic pathology, potentially reducing diagnostic accuracy in those regions [19]. Another potential limitation is that filtered phase images may exhibit aliasing artifacts in regions with significant or extensive calcification, which can complicate the precise evaluation of the lesion’s characteristics and size [81]. Finally, considering that SWI has a slightly longer acquisition time than T2*, it may be more susceptible to motion artifacts, especially in less cooperative patients (e.g., children, Figure 9B).

To mitigate blooming artifacts, a quantitative method such QSM, which generates a quantitative map of magnetic susceptibility, can be used. Furthermore, QSM enables easy discrimination between calcification and hemorrhage because it renders their magnetic susceptibility differences as low- and high-contrast regions, respectively [2]. The QSM artifact reduction technique (QSMART) is a post-processing technique that constructs susceptibility maps from phase images, aiming at reducing artifacts in areas with significant susceptibility differences, as evaluated in humans using 7 Tesla MRI scans. This leads to a decrease in streaking artifacts and the removal of banding artifacts on the cortical surface and near blood vessels [82].

## 6. Conclusions

SWI is an MRI sequence that offers valuable insights into the presence and distribution of paramagnetic and diamagnetic substances within the brain. Clinically, it is the most sensitive technique for identifying microhemorrhages and iron deposits, the patterns of which are crucial for assessing neurodegenerative disorders. SWI is increasingly used in MRI protocols for these conditions due to its ability to detect neuroimaging markers, some of which are now included in diagnostic criteria. The principles outlined in this review can assist radiologists in leveraging the full potential of SWI, while remaining mindful of its limitations and technical nuances. Looking ahead, radiomics and QSM will likely play an increasingly central role in the clinical application of SWI, offering new opportunities for tissue characterization, quantitative analysis, and the development of imaging biomarkers, establishing the way for more personalized and data-driven approaches in neuroimaging.

## Figures and Tables

**Table 1:** Summary of the main SWI findings and signs for each neurodegenerative disease. MSA-P: multiple system atrophy—parkinsonian subtype; PSP: progressive supranuclear palsy.

Neurodegenerative Diseases	SWI Findings	Description
Parkinson’s disease, Lewy body dementia, and atypical parkinsonian syndromes	Absent swallow-tail sign	Loss of the normal bright signal in the posterior third of the substantia nigra (nigrosome-1)
	Iron deposition	MSA-P: hypointensity in the putamen PSP: hypointesity in basal ganglia, red nucleus, substantia nigra pars reticulata, and cerebellar dentate nucleus
Multiple sclerosis	Central vein sign	Punctate or linear hypointensity at the center of a hyperintense lesion in at least 2 of 3 orthogonal planes (>2 mm)
	Paramagnetic rim lesions	Hypointense rim surrounding an internal lesion that is isointense to adjacent normal white matter
Cerebral amyloid angiopathy	Cortical or cortico–subcortical microbleeds	Small (2–10 mm), multiple (≥2), round or ovoid, and uniformly hypointense, primarily located in the frontal and parietal lobes (usually sparing the basal ganglia, assisting in the differential diagnosis with hypertensive microangiopathy)
	Convexity subarachnoid hemorrhage/cortical superficial siderosis	Curvilinear regions of signal drop-out localized to one or more sulci
Amyotrophic lateral sclerosis	Motor band sign	Curvilinear bands of reduced signal in the gray matter of the primary motor cortex
Hereditary ataxias	Abnormal dentate nuclei	Atrophy in spinocerebellar ataxia 6, Friedreich’s ataxia, and spinocerebellar ataxia 3; Decreased iron concentration in oculomotor apraxia
Huntington’s disease	Iron deposition	Hypointesity in the basal ganglia (mainly globus pallidus)
Neurodegeneration with brain iron accumulation	Eye of the tiger sign	Symmetric bilateral abnormal low signal in the globus pallidus with central high signal
Chronic traumatic encephalopathy	Diffuse axonal injury (microbleeds)	Punctate or linear hypointensity at the gray–white matter junction, in the corpus callosum or the brainstem

## Abbreviations

AD	Alzheimer’s disease
BSI	blood-sensitive imaging
CAA	cerebral amyloid angiopathy
CVS	central vein sign
FLAIR	fluid-attenuated inversion recovery
FSBB	flow-sensitive black blood
GRE	gradient recalled echo
MinIP	minimum intensity projection
MRI	magnetic resonance imaging
MS	multiple sclerosis
MSA	multiple system atrophy
PD	Parkinson’s disease
PSP	progressive supranuclear palsy
QSM	quantitative susceptibility mapping
QSMART	QSM artifact reduction technique
SWAN	susceptibility-weighted angiography
SWI	susceptibility-weighted imaging
SWI-p	susceptibility-weighted imaging-phase
TE	echo time
TR	repetition time
